# Printing Beyond Fabrication: A Physicochemical Vision for Emerging Device Technologies

**DOI:** 10.1021/acsphyschemau.6c00089

**Published:** 2026-07-15

**Authors:** Naimeh Naseri

**Affiliations:** † Department of Electrical and Electronic Engineering, RMIT University, Melbourne, Victoria 3001, Australia; ‡ Department of Mechanical and Aerospace Engineering, 2541 Monash University, Clayton, Victoria 3168, Australia; § Chair of Analytical Chemistry II, Faculty of Chemistry and Biochemistry, Ruhr University Bochum, Universitätsstraße 150, Bochum 44801, Germany

**Keywords:** printable devices, supply chain, interface science, sustainable printing platforms, science-driven manufacturing

## Abstract

The future of printing cannot be understood as the evolution of another manufacturing tool. Rather, it points to a science-driven paradigm in which functional matter is designed, formed, and integrated through controlled physicochemical events. In this visionary article, we look back to show how advances in nonlinear photochemistry, nanoscale heat transport, capillary flow, electrohydrodynamics, and liquid metal interfacial science have matured into powerful printing platforms, exampling two-photon polymerization, meniscus-guided printing, and liquid metal electronics. These histories reveal that transformative printing emerges when physical chemistry turns instability, confinement, reaction thresholds, surface forces, and energy gradients into manufacturing principles. Looking forward, printing may democratize smart systems by enabling localized, digital, adaptive, and on-demand fabrication of electronics, sensors, energy devices, and wearable technologies, while also supporting more resilient supply chains by reducing dependence on centralized manufacturing and expanding access to functional technologies closer to the point of need. Yet this future requires solving challenges in predictive ink design, solution-free material formation, durability, integration, circularity, standardization, and AI-guided control. Physical chemistry will be central to making printing reliable, sustainable, and widely accessible.

## Graphical abstract

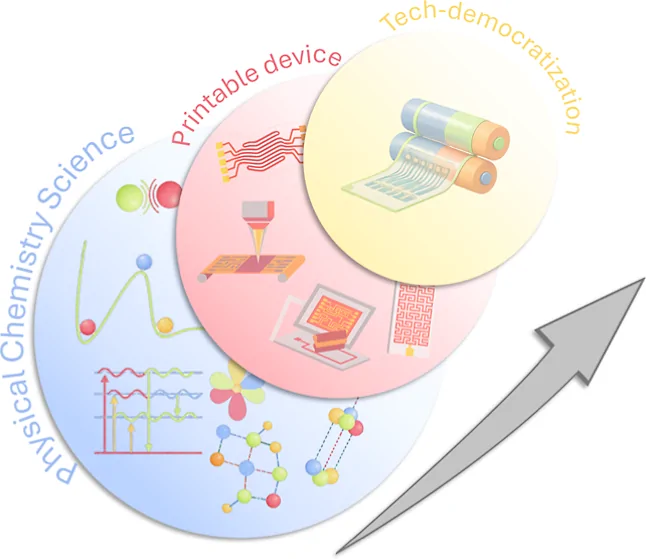

## 1 ■ Printing: a Paradigm Shift in Supply Chains

Long before printable electronics became part of the scientific imagination, the printing press showed that printing could change the world. What began as a way to reproduce text became one of the most powerful forces in human history, reshaping education, literacy, science, religion, politics, and commerce.^1^ At the time, few could have imagined that making words easier to reproduce would help redraw the social and economic map of civilisation. Yet this was the deeper power of printing: it did not merely make books; it democratised access to knowledge.^2,3^

Today, we may be standing at the edge of another printing revolution, one that moves beyond the book. Printable electronics, sensors, and energy devices could do for functional technologies what the printing press once did for knowledge: shift them from centralized, specialized, and expensive systems toward more distributed, customizable, and widely accessible platforms.^4^ The past decade has already witnessed a rapid expansion of printable devices, driven by advances in materials design and scalable fabrication strategies. These systems are now reaching across healthcare diagnostics, wearable monitoring, IoT networks, energy harvesting, and smart packaging, signaling a future in which printing is no longer just a fabrication route, but a science-driven platform for democratising smart technologies.^5^

Conventional manufacturing of electronic devices has been historically anchored in centralized, capital-intensive infrastructures optimized for scale but inherently rigid, resource-intensive, and slow to adapt. Complex, multistep fabrication routes—spanning material preparation, deposition, patterning, assembly, and encapsulation—not only introduce inefficiencies in material usage but also constrain rapid design iteration and functional integration. This model is tightly coupled to a globally distributed supply chain, where upstream material processing, midstream component fabrication, and downstream system integration are geographically separated and logistically interdependent. While this structure has enabled mass production, it remains vulnerable to disruptions, delays, and rising environmental and economic costs, particularly as demand grows for more adaptive, multifunctional, and application-specific electronic systems.

Printing technologies introduce a fundamentally different manufacturing logic that directly challenges this paradigm. Rather than assembling prefabricated components through sequential processes, printing can enable the direct fabrication of functional devices from digitally defined inputs, with precise control over material placement and structure. This approach has the potential to consolidate multiple fabrication steps into a unified process, reducing complexity, material waste, and production time while enabling rapid customization without extensive retooling. More importantly, it decouples device fabrication from centralized infrastructure, allowing production to be distributed, localized, and increasingly responsive to specific application needs, thereby lowering barriers to entry and enabling broader access to advanced device technologies across diverse geographic and economic contexts. This shift supports the democratization of device manufacturing, where design, production, and deployment are no longer confined to specialized facilities but can be extended to smaller-scale, resource-limited, and point-of-use environments.

This shift has profound implications for the structure of electronics supply chains. As schematically illustrated in Figure 1, the conventional linear model—characterized by upstream material sourcing, centralized manufacturing and assembly, and downstream distribution through physical warehouses—is progressively being reconfigured into a more distributed and digitally mediated network. In this emerging paradigm, functional materials still originate upstream, but fabrication moves closer to the point of use through localized manufacturing hubs. Design and inventory are increasingly digitized, residing in “digital warehouses” that enable on-demand production based on validated, application-specific templates. This transformation reduces reliance on long-distance logistics, minimizes inventory burdens, and enables more agile and resilient responses to supply chain disruptions. At the same time, printing facilitates decentralized and on-demand fabrication, where devices can be produced closer to end users or integrated directly into target systems. This not only lowers transportation-related costs and emissions but also enables new modes of device integration, where electronics are embedded into structures, surfaces, and everyday materials rather than assembled as discrete components. The convergence of printing with automation, robotics, and data-driven control further reinforces its role within smart manufacturing ecosystems, enabling adaptive production environments that can respond dynamically to changes in design, material availability, and performance requirements. Equally significant is the alignment of printing with sustainable and circular manufacturing principles. By depositing materials only where needed, printing can minimize waste compared to conventional subtractive or overcoating processes. Its compatibility with reformulated and recycled functional materials further opens pathways toward closed-loop production, where fabrication and material recovery are more tightly integrated. In this context, printing is not simply an alternative fabrication technique but a potential structural enabler of a new supply chain paradigm—one that is distributed, digitally driven, and inherently more resilient, adaptable, and sustainable.

**Figure 1:**
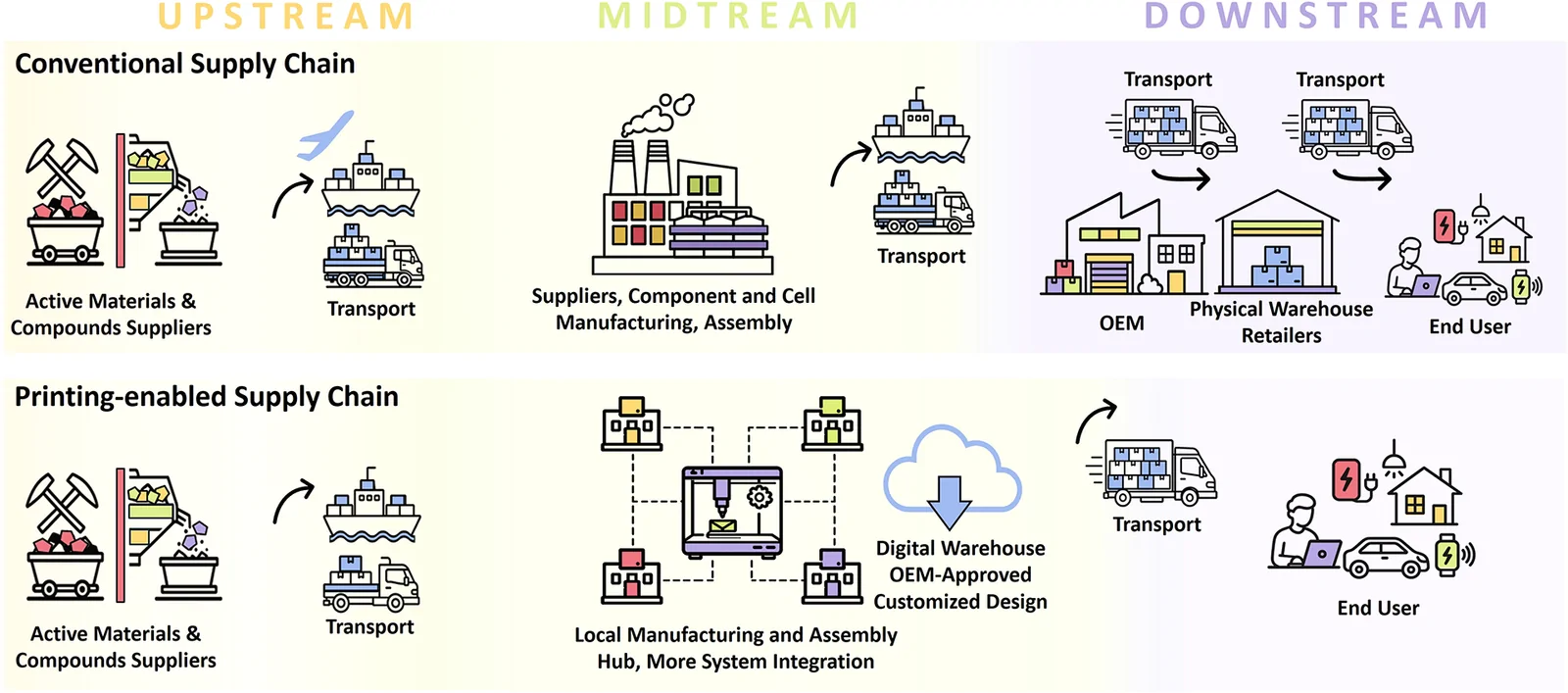
Conventional vs printing-enabled supply chains for electronic devices. Top: centralized model with upstream suppliers, midstream manufacturing, and downstream distribution to OEMs (original equipment manufacturers), warehouses, and retailers, relying on transport-intensive logistics before reaching end users. Bottom: printing-enabled model with OEM-approved digital designs stored in “digital warehouses” and fabricated on demand in localized hubs near the point of use, potentially reducing transport needs, minimizing physical retail dependence, and enabling a more distributed, accessible, and responsive supply chain. Here, end-of-life recovery and recycling pathways are not shown but remain essential to a complete circular printing-enabled supply chain.

Although the transition from centralized manufacturing toward digitally connected, distributed production remains at an early stage for printable electronics and functional devices, emerging developments in additive and smart manufacturing provide a glimpse of how such a transformation may unfold. A techno-economic assessment of 94 manufacturing sectors identified a wide range of products—from medical instruments to sports goods—as suitable candidates for additive-manufacturing-enabled distributed production.^6^ Importantly, computer and communication equipment were ranked among the top 20 sectors, suggesting that elements of distributed manufacturing may also become increasingly relevant for future electronic-device supply chains.

This logic has been further explored in the automotive spare-parts sector. In a Volvo Group case study,^7^ 22 selected spare parts for the Australian market were analyzed under a scenario in which parts were produced locally by additive manufacturing rather than manufactured in Europe and transported through central and regional distribution warehouses before reaching Australian dealers. The analysis estimated that localized additive manufacturing could reduce average transport distance by approximately 90–92% and lead time by 91–97%. Beyond these numerical gains, the case illustrates how digital part files, local production capacity, and on-demand fabrication can reduce dependence on long logistics chains and improve responsiveness for low-volume or unpredictable spare part demand.^7^

Lessons from COVID-19 further show how printable formats can broaden access when deployment barriers are reduced across health systems. Lateral-flow diagnostic strips moved testing beyond centralized laboratories into homes, schools, workplaces, borders, and community settings, with more than three billion COVID-19 tests conducted globally.^8^ Their low cost, rapid readout, and suitability for self-testing supported unprecedented scale, including international procurement efforts that negotiated antigen-test price ceilings of approximately US$2.50 and distributed more than 158 million tests. Despite persistent inequities in access, this example demonstrates how printable devices can help democratize functional technologies and points to the need for more distributed, affordable, and equitable production–deployment models.

## 2 ■ The Landscape of Printable Devices

Printing is often described as a deposition technique, but in printable devices, it is better understood as a physicochemical route to functional architecture. A printed feature is not simply placed on a surface; it emerges through the coupled control of composition, rheology, transport, wetting, confinement, assembly, transformation, and integration.^9,10^ These pillars define how an ink flows, spreads, dries, reacts, organizes, adheres, and ultimately connects with other printed layers to produce device function. At the heart of this landscape are the core principles of physical chemistry (Figure 2). Thermodynamics governs phase stability, miscibility, surface energy, and the driving forces for wetting, crystallization, segregation, and self-assembly. Kinetics controls the rates of jetting, evaporation, diffusion, particle migration, curing, reaction, and structural locking. Interfacial science determines how inks meet substrates, how contact lines move or pin, how layers adhere, and how charge, ions, or molecules cross material boundaries. Seen through this lens, printing is not a passive manufacturing step but a dynamic, nonequilibrium transformation of matter under flow, confinement, evaporation, and interfacial contact. The future of printable devices will therefore be shaped not only by better printers, but by a deeper ability to program these physicochemical processes into reliable, scalable, and multifunctional device architectures. To make this connection explicit, Table 1 maps representative printing variables onto the physicochemical mechanisms and device-level outcomes that they control.

**Figure 2:**
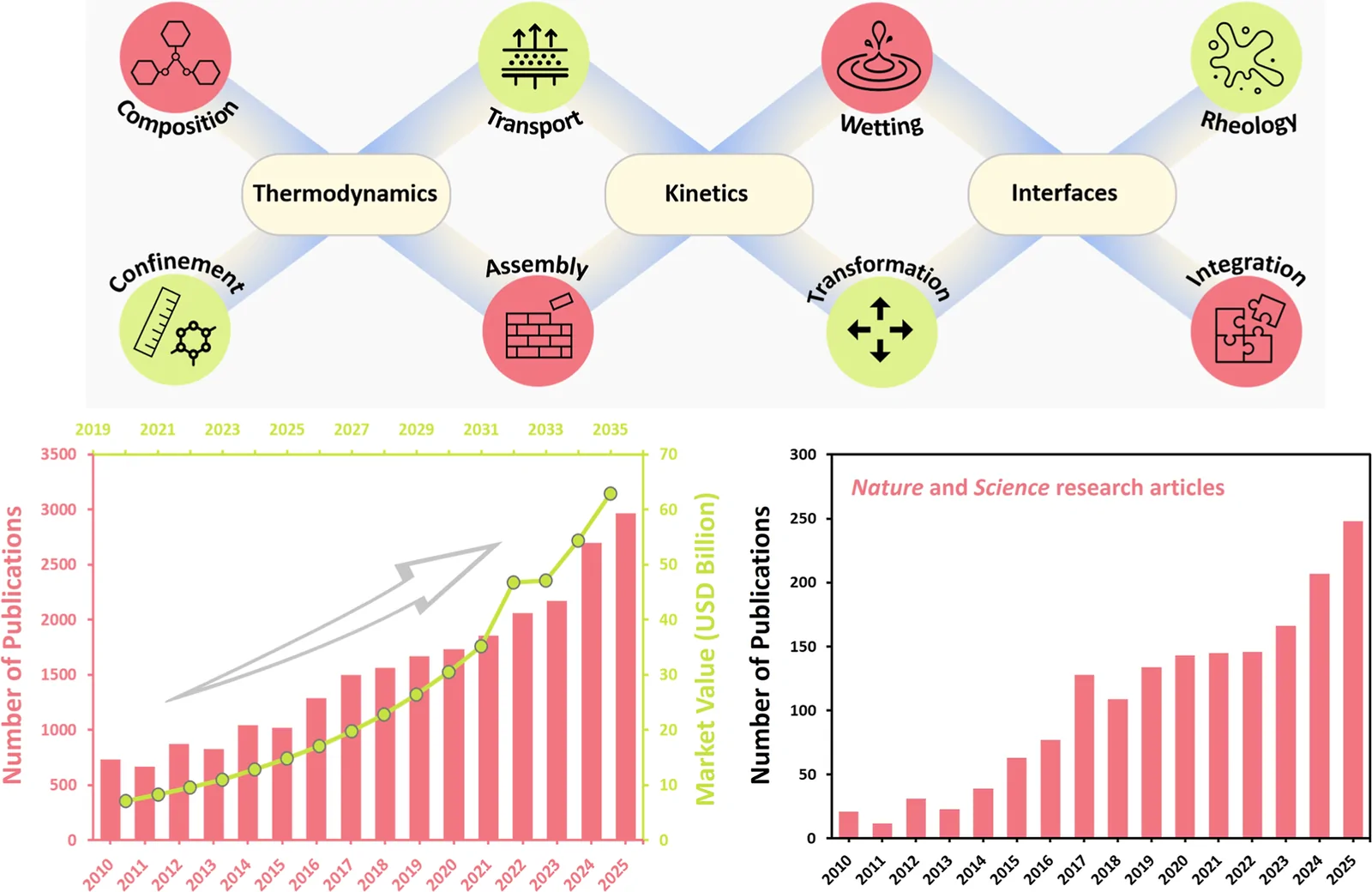
Evolving landscape of printable devices and its physicochemical foundations. Top: schematic illustrating the interconnected pillars of printable device technologies and their strong coupling to core physical chemistry domains, namely thermodynamics, kinetics, and interfacial science. Bottom left: growth of the research and industrial landscape based on Scopus data obtained using the keywords “print*” AND “electronics” in TITLE-ABSTRACT-KEYWORDS, including all indexed publication types, accessed April 20, 2026, together with reported and projected market values.^8^ Bottom right: number of research articles published in Nature journals (main family only, excluding npj titles and review papers) and Science journal, showing the growing presence of leading fundamental contributions in the printable device field.

**Table 1:** Conceptual Framework Linking Physical Chemistry Principles to Printing-Relevant Variables and Device-Level Outcomes

physicochemical foundation		representative printing variables	governing mechanism	representative outcomes
Thermodynamics	composition	solvent composition, concentration, additive selection, energy matching	solvation, phase stability, intermolecular interactions, free-energy minimization	stable inks, uniform distribution, conductivity, device stability, response, activity
	confinement	feature dimensions, layer thickness, droplet volume, voxel size	confinement-induced phase behavior, localized energy landscapes	nano/microscale structure control, integration density, sensitivity, switching behavior
kinetics	transport	printing speed, drying rate, temperature, energy input	heat and mass transfer, diffusion, evaporation kinetics	uniform deposition, solidification, uniformity, defect density, reproducibility
	assembly	deposition rate, particle loading, curing profile	crystallization, particle assemblies, growth dynamics	tailored microstructure, connectivity, charge transport
	transformation	exposure dose, reaction time, thermal budget, activation conditions	polymerization, cross-linking, phase transformation, chemical conversion	functional material formation, pattern stabilization, mechanical robustness
interfaces	wetting	surface energy, substrate treatment, droplet spacing	capillarity, spreading, contact-line dynamics	pattern fidelity, dimensional accuracy, resolution, line-edge quality
	rheology	viscosity, viscoelasticity, shear rate, yield stress	flow behavior under confinement and applied stress	printing stability, process reliability, feature uniformity, scalability
	integration	layer sequence, adhesion strength, multimaterial compatibility	interfacial bonding, charge and mass transfer across interfaces	multifunctionality, device integration, flexibility, architectural complexity

The rise of printable devices is now visible across both the research literature and the industrial landscape, pointing to a field that is no longer defined only by fabrication convenience but by its scientific depth and technological reach. As shown in Figure 2, publications related to printable electronics have grown markedly over the past decade, while the market is projected to expand from approximately USD 14.8 billion in 2025 to USD 62.9 billion by 2035, corresponding to a CAGR of 15.6%.^11^ This parallel growth in academic output and commercial demand reflects a field entering a more mature phase, where progress depends on the close coupling of fundamental insight and application-driven design. Notably, the increasing appearance of printable-device research in leading journals such as Nature and Science families is more than a marker of visibility. It suggests that the most influential advances in this area are often science-demanding breakthroughs, not simply demonstrations of improved manufacturing. Many of these studies tackle fundamental questions in interfacial dynamics, fluid transport, confined reactions, soft-matter assembly, phase transformation, and nonequilibrium materials formation. In this sense, the publication trend mirrors a deeper shift in the identity of printing: from a tool for patterning materials to a platform for discovering, controlling, and translating physicochemical phenomena into functional devices.

## 3 ■ Physicochemical Origins of Breakthrough Technologies

Breakthroughs in printable technologies are not accidental advances in fabrication—they are the direct manifestation of deeper progress in physical chemistry. What appear today as sophisticated printing capabilities are, in essence, the translation of earlier insights into transport processes, surface interactions, and dynamic materials conversion into controllable manufacturing processes. In a rapidly expanding landscape, it is this continued evolution of physicochemical understanding that is shaping the next generation of printing technologies, as can be seen in several recent breakthrough examples. Even within traditionally lithographic and partly subtractive methods, science-driven breakthroughs continue to align with the central message. Hence, they are included here as an opening case study, as they exemplify physicochemically driven patterning paradigms that have evolved alongside additive printing and continue to play an important role in advanced device fabrication.

### 3.1 From Nanoscale Heat Transport to Functional Nanofabrication

In a pioneering 1992 work by IBM,^12^ H. J. Mamin and D. Rugar introduced thermomechanical writing using a heated atomic force microscope tip, where localized heat transfer softens a polymer surface and enables nanoscale indentation under controlled load (Figure 3A­(i,ii)). They demonstrated feature sizes on the order of ∼100 nm, establishing a route toward high-density patterning; however, resolution and scalability were still limited by heat diffusion, tip geometry, and the inherently serial nature of probe-based writing. While alternative approaches soon emerged—such as dip-pen nanolithography based on capillary-driven molecular transport (∼30 nm resolution)^13^ and imprint lithography exploiting thermoplastic flow and molding (∼25 nm resolution)^14^—thermally driven scanning probe methods continued to attract attention due to their unique ability to directly couple nanoscale heat transport, phase transformation, and mechanical actuation, offering unmatched control over local material modification beyond purely chemical or replication-based patterning.^15–20^ This approach matured in later work, where thermally written nanoscale patterns were coupled with multilayer transfer stacks and selective etching to achieve sub-20 nm feature sizes in silicon and metal structures, effectively transforming thermal scanning probe lithography (t-SPL) from a surface modification technique into a viable nanofabrication platform.^21^

**Figure 3:**
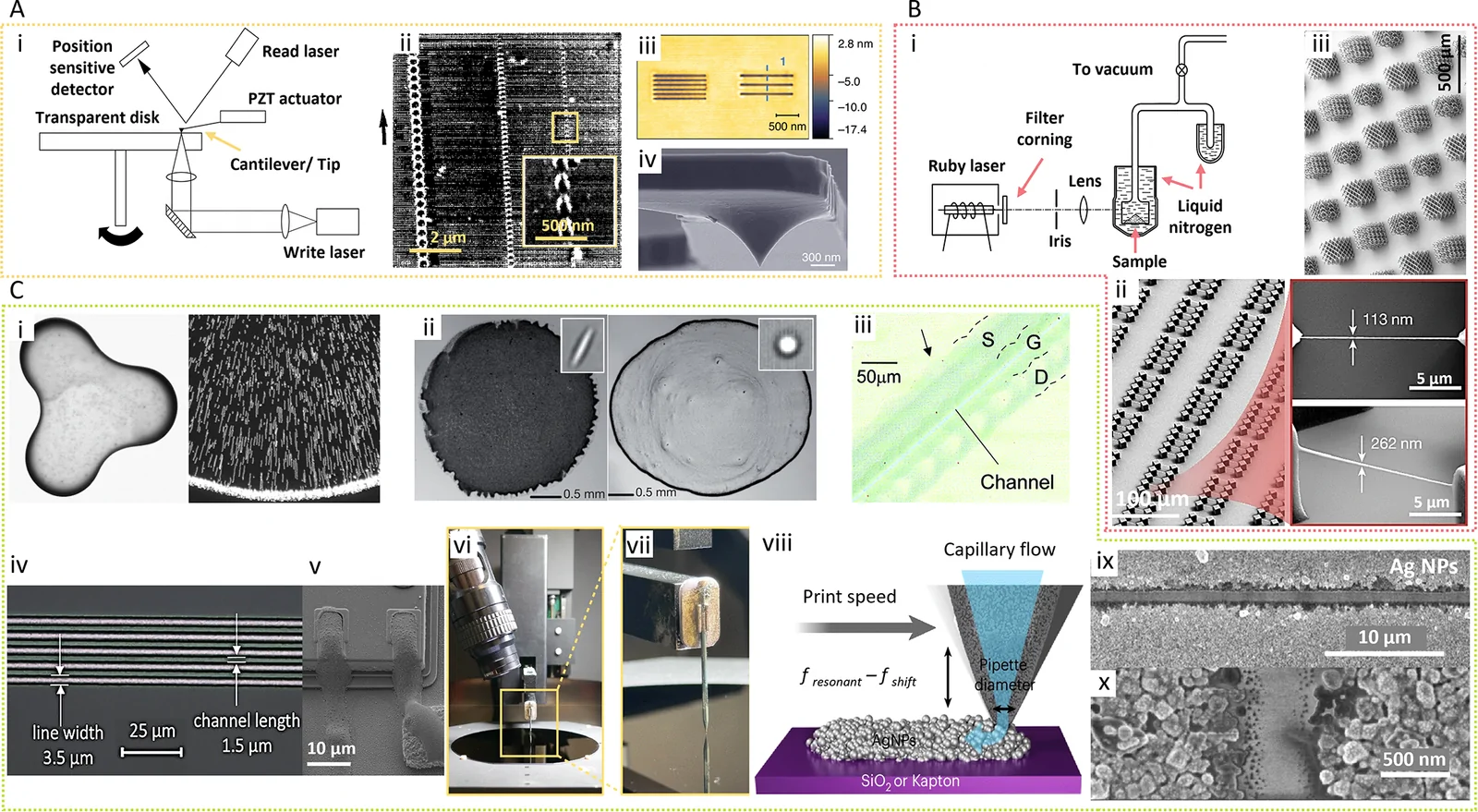
(A) (i) The original IBM concept for AFM-tip-based thermomechanical writing and (ii) the resulting nanoscale written pattern. Reproduced with permission from.^12^ Copyright 1992 AIP. (iii) State-of-the-art t-SPL/NanoFrazor patterning. Reproduced from.^20^ Available under a CC-BY 4.0 license. Copyright 2026 Li et al. (iv) SEM image of the used cantilever tip in modern NanoFrazor systems. Reproduced from.^19^ Available under a CC-BY 4.0 license. Copyright 2024 Erbas et al. (B) (i) Early apparatus used for laser-induced photopolymerization experiments. Reproduced with permission from.^23^ Copyright 1965 AIP. (ii) State-of-the-art metalens-enabled two-photon printing, and (iii) printed 3D microlattice cubes. Adapted with permission from.^27^ Copyright 2025 Springer Nature. (C) (i) Original coffee-stain study showing ring-like deposition in a dried 2 cm coffee droplet (left) and particle accumulation at the high-curvature edge region (right). Adapted with permission from.^33^ Copyright 1997 Springer Nature. (ii) Dried droplet uniformity dependence on particle shape (inset). Adapted with permission from.^35^ Copyright 2011 Springer Nature. (iii) Inkjet-printed thin film transistor. Adapted with permission from.^36^ Copyright 2000 AAAS (iv) Optical images of the UPD-printed electrodes. Reproduced from.^42^ Available under a CC-BY 4.0 license. Copyright 2023 Losi et al. (v) Printed high-quality microIC interconnections by XTPL technology. Reproduced from.^40^ Available under a CC-BY 4.0 license. Copyright 2025 Waldner et al. (vi) Photo of HPCaP (NAZCA) printing platform’s pipet, (vii) in contact with substrate during capillary writing, (viii) schematic of AgNP ink deposition, and (ix,x) SEM images of the resulting printed patterns and preserved gaps. Adapted with permission from.^44^ Copyright 2025 Springer Nature.

Recent advances built on the NanoFrazor platform highlight the technical maturation of t-SPL through both materials and process engineering (Figure 3A (iii,iv)). The introduction of thermally responsive resists such as PPC—governed by controlled pyrolysis via chain unzipping and random scission—enables high-fidelity grayscale patterning (∼50 nm, subnm depth control).^20^ Additionally, optimized dry etching allows shallow t-SPL-written polymer patterns to be transferred into dielectric nanostructures with ∼5–10 × depth amplification while preserving shape fidelity.^19^ Beyond fabrication, the same physicochemical principles have recently enabled t-SPL to evolve into a nanoscale probing platform.^17^ By exploiting localized periodic heating and temperature-wave analysis, the technique uses the probe as a confined heat source (∼10–100 nm) and extracts thermal diffusivity from the phase delay of heat propagation governed by diffusion kinetics and thermal diffusivity contrasts. This shift—from detecting temperature to actively driving and decoding heat transport at the nanoscale—demonstrates how advances in heat transfer physics and dynamic thermodynamics extend t-SPL from material transformation to quantitative thermophysical characterization. This evolution extends to device fabrication, where t-SPL leverages nanoscale heat transfer and resist decomposition kinetics to directly define functional silicon nanowire transistors with high-dimensional control.^22^ Although rooted in lithographic and largely subtractive paradigms, these approaches remain among the most powerful strategies for nanoscale patterning, as they leverage precise control over heat transport to define structures with unmatched fidelity.

### 3.2 Nonlinear Photochemistry to Programmable 3D Printing

Two-photon polymerization stands as a compelling example of how advances in physical chemistry translate into new fabrication paradigms. Its origin can be traced to early explorations of nonlinear light–matter interactions, where Yoh-Han Pao and P. M. Rentzepis first demonstrated laser-induced radical generation and polymerization under conditions inconsistent with single-photon absorption in 1965, pointing to multiphoton—most likely two-photon—processes (Figure 3B­(i)).^23^ This foundational insight established that chemical reactivity can be governed by photon density and nonlinear excitation pathways. Decades later, this principle evolved into a controllable platform through the molecular design of photoinitiators with large two-photon absorption cross sections, dramatically lowering polymerization thresholds and translating nonlinear excitation into practical three-dimensional fabrication.^24^ A decisive turning point was reached in 2001,^25^ when the voxel size was no longer dictated by the optical focal spot but by a reaction threshold arising from the interplay of two-photon absorption and radical quenching, enabling feature sizes down to ∼120 nm—well below the diffraction limit. This established that spatial resolution is fundamentally governed by nonlinear reaction kinetics and threshold behavior rather than optical constraints alone. These advances mark a progression from the discovery of multiphoton-induced chemistry to the deliberate control of reaction pathways and spatial confinement, setting the stage for the next generation of breakthroughs built upon this physicochemical foundation.

Today, advancements illustrate how this deeper physicochemical control is now being translated into scalable printed architectures. One pathway emerges from light-sheet 3D microprinting via two-color two-step absorption, where polymerization is governed by a kinetic “AND gate” requiring sequential excitation through intermediate states.^26^ By decoupling excitation pathways and exploiting excited-state lifetimes, this strategy achieves tight spatial confinement without extreme intensities and simultaneously overcomes the traditional resolution–throughput trade-off, enabling submicrometre voxels at multimillion voxel-per-second rates. In parallel, a distinct breakthrough redefines scaling at the system level through metalens-based parallelization, where meta-optics and programmable light modulation generate more than 10^5^ independently controlled focal spots, enabling centimeter-scale, stitch-free fabrication at >10^8^ voxels s^–1^ while preserving sub-100 nm features.^27^ Together, these advances build on earlier progress in simply localizing reactions—an approach that underpinned technologies such as Nanoscribe^28^—and extend it toward high-resolution nanomanufacturing (Figure 3B­(ii,iii)).

Recent advances in 3D printing show how far the field has moved beyond shape-making. Embedded printing has enabled fully integrated hydrogel electronics, where soft, stretchable structures and conductive pathways are formed within the same compliant material system.^29^ Nonlinear photopolymerization has pushed 3D microfabrication to the level of device-ready architectures, including high-density microelectrodes directly integrated with silicon electronics.^30^ Charge-programmed 3D fabrication has further expanded the design space by using electrostatic patterning to guide selective, multimaterial growth inside complex geometries.^31^ Together, these examples show printing becoming a platform for programming structure, function, and integration in three dimensions. Yet, their strengths also reveal their limits: many remain dependent on specialized instruments, narrow material windows, and modest throughput. This gap points to the next frontier—fluid- and ink-based printing strategies that can translate precision and functionality into scalable, material-diverse, and more accessible manufacturing.

### 3.3 From Interfacial Fluid Physics to Deterministic Liquid Printing

The evolution of liquid-based printing and coating technologies is deeply rooted in advances in interfacial physical chemistry, where early efforts to understand liquid behavior at interfaces gradually transformed uncontrolled phenomena into programmable processes. Foundational insights dating back to the Marangoni effect^32^ established that surface tension gradients—arising from temperature or concentration differences—can drive fluid motion at interfaces. Decades later, this fundamental principle was embedded into evaporating systems through the seminal work of Robert D. Deegan and co-workers,^33^ who revealed that the ubiquitous “coffee-ring” pattern originates from evaporation-driven capillary flow under pinned contact lines, where outward transport concentrates material at the droplet edge (Figure 3C­(i)). This work turns an everyday artifact into a predictable transport mechanism, and subsequent studies expanded this framework from description to control. Later work showed that the coffee-ring effect is not unavoidable. Marangoni flows, generated by evaporation-induced surface-tension gradients, can counter the outward capillary transport and redirect material toward the droplet center.^34^ A complementary advance came from interfacial design: anisotropic particles can distort the liquid–air interface, create strong capillary interactions, and suppress ring formation without changing the solvent or drying conditions (Figure 3C­(ii)).^35^ Together, these studies shifted the field from simply observing drying stains to actively controlling deposition, providing a key foundation for modern inkjet and drop-on-demand printing of functional materials.

Building on this foundation, early inkjet-based studies demonstrated how interfacial control could be translated into practical patterning strategies. In a seminal contribution, Sirringhaus et al.^36^ showed that high-resolution transistor architectures could be achieved not by reducing droplet size, but by engineering surface energy landscapes to confine droplet spreading (Figure 3C­(iii)). A decade later, this concept was extended further, where inkjet printing was combined with antisolvent-induced phase transformation to control crystallization within droplets, enabling the direct formation of highly uniform organic single-crystal films at the liquid–air interface.^37,38^

Today, ultraprecise deposition (UPD) technologies—exemplified by XTPL—translate evaporation-driven transport, capillary flow, and meniscus stability into deterministic writing tools.^39–41^ Emerging from advances in microfluidics and interfacial physics, UPD departs from droplet-based inkjet by enabling continuous, capillary-fed submicrometre deposition. The technology demonstrates that UPD can directly print submicrometre metal contacts, enabling high-frequency organic transistors, overcoming the long-standing resolution limits of solution-based printing^41^ and having device-level performance traditionally accessible only by lithography.^42^ By engineering surface energy at multimaterial interfaces, the capillary spreading and short-circuiting limits in printing across 3D chip topographies are avoidable now, with reliably bridged microIC–microLED architectures for next-generation microLED display technology, without conventional backplane fabrication (Figure 3C­(iv,v)).^40^

As another emerging approach, High Precision Capillary Printing (HPCaP), as developed by HUMMINK,^43^ is based on capillary-mediated ink transfer from an ink-filled glass pipet to the substrate, rather than discrete droplet ejection or pressure-driven dispensing. Inspired by AFM-style probe positioning, the system uses capillary forces and piezo-driven resonance to stabilize the liquid bridge formed between the pipet and substrate. This offers highly localized deposition with reported resolutions down to ∼100 nm and compatibility with inks spanning a broad viscosity range (Figure 3C­(vi–x)). Accordingly, a printed submicrometre carbon nanotube transistor (channel lengths down to ∼500 nm) without chemical patterning or postfabrication structuring is demonstrated, overcoming the long-standing resolution limit (∼10–30 μm) of conventional printing methods and by stabilizing meniscus-driven deposition and eliminating droplet-related instabilities (e.g., overspray, satellite drops)·^44^

### 3.4 Field–Fluid Coupling in Electrohydrodynamic Printing

Electrohydrodynamic (EHD) printing is rooted in field–fluid coupling, where electric stresses act directly on liquid interfaces, converting electrostatic energy into fluid motion and jet formation.^45^ In an early foundational study, Crowley demonstrated that applying an electric field to a liquid jet introduces an electric pressure term (∝ εE^2^) that perturbs and destabilizes the jet in a controlled manner, enabling predictable droplet formation governed by a balance of inertia, surface tension, and electrostatic forces.^46^ Complementing this, the Nature study uncovered the interfacial transport physics underlying stable jet formation, showing that EHD systems are governed not just by normal electric pressure but by tangential electrical shear stresses (τ ∼ ε_0_E_n_E_t_) acting on charged interfaces (Figure 4A­(i)).^47^ This introduces a fundamentally different transport mechanism: charge-driven interfacial convection, where surface charge gradients and electric fields generate internal circulation within the Taylor cone. The result is a nonuniform velocity field—fast at the interface, reversed at the core—stabilizing and thinning the jet. Later in 2007, scientists could overcome the coarse and unstable nature of prior EHD jetting by demonstrating controlled, submicrometre e-jet printing, driven by refined nozzle design and precise tuning of jet modes (Figure 4A­(ii)).^48^ By harnessing the transition between pulsating and stable regimes, it transformed EHD from an uncontrolled spraying phenomenon into a deterministic, device-capable direct-write technique.

**Figure 4:**
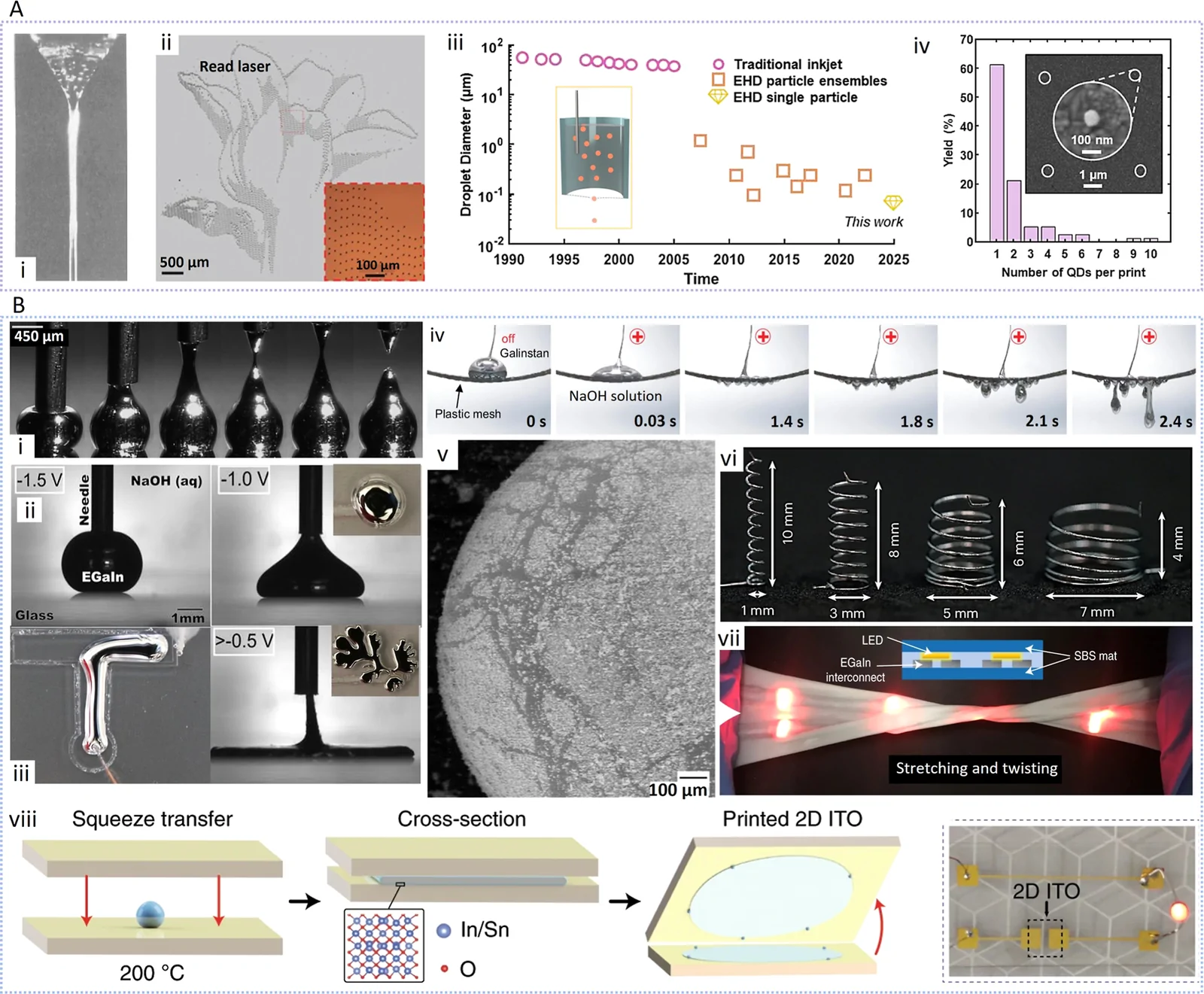
(A) (i) Original visualization of lycopodium particles within the conical base of a stable EHD jet, adapted with permission from.^47^ Copyright 1986 Nature, (ii) followed by later EHD printing of SWNT dot patterns, adapted from.^48^ Available under a CC-BY 4.0 license. Copyright 2007 Losi et al. (iii) Recent single-particle EHD printing compared with other printing technologies developed over the past 35 years, and (iv) a representative histogram of printed quantum dots. Adapted with permission from.^50^ Copyright 2026 Wiley. (B) (i) A conical tip formation in EGaIn. Adapted with permission from ref 53. Copyright 2008 Wiley. (ii) Oxidative spreading of a bead of liquid metal in 1 M NaOH solution at different applied potentials, and (iii) by introducing to a capillary channel. Adapted from.^56^ Available under a CC-BY 4.0 license. Copyright 2014 National Academy of Sciences. (iv) Penetration of liquid metal into plastic mesh by applying voltage. Adapted from.^57^ Available under a CC-BY 4.0 license. Copyright 2020 Yun et al. (v) A galinstan droplet encapsulated by WO_3_ nanoparticles as a liquid metal marble. Adapted with permission from.^55^ Copyright 2013 Wiley. (vi) Photo of 3D printed free-standing helical metal structures. Adapted with permission from.^62^ Copyright 2024 Springer Nature. (vii) An encapsulated stretchable mat printed with EGaIn. Adapted with permission from.^60^ Copyright 2021 Springer Nature. (viii) Squeeze printing of an indium–tin alloy to form ultrathin ITO sheets, followed by patterning into a circuit capable of powering an LED. Adapted with permission from.^59^ Copyright 2021 Springer Nature.

After the early foundations of EHD jetting, the field has now reached a stage where proof-of-concept demonstrations are beginning to look like real manufacturing capabilities. One example is the EHD printing of ligand-engineered MXene inks, where chemically stabilized 2D nanosheets can be assembled into well-aligned, densely packed planar films while retaining their morphology, conductivity, and environmental stability.^49^ This shows the strength of EHD as a route for processing difficult functional inks into device-relevant structures. Yet the process still operates through collective flake assembly: it can organize ensembles of nanosheets but not yet place or orient individual flakes on demand. A more radical step is emerging through single-particle extraction electrohydrodynamics (SPEED), where EHD printing moves beyond droplet or jet delivery into deterministic nanoscale placement.^50^ Instead of ejecting a bulk fluid volume, dielectrophoretic forces extract and position individual quantum dots by overcoming single-particle interfacial energy barriers. Operating at subzeptolitre volumes, this approach sharply reduces stochastic deposition and material waste, offering single-photon-emitting quantum dots to be integrated with nanophotonic and quantum device architectures (Figure 4A­(iii,iv)). Together, these advances signal a broader transition for EHD printing from assembled thin films to individual nanoscale emitters. The next frontier is to turn this precision into a manufacturable language for scalable, material-diverse printed systems.

### 3.5 Liquid and Printable Metals from High-Energy Fluids to Programmable Platforms

Liquid metals have long been known to humanity, with mercury recognized since antiquity and used across centuries in applications ranging from thermometers and barometers to amalgamation in metallurgy and early medicinal practices. Despite this long-standing familiarity, systematic understanding of their behavior emerged much later through developments in physical chemistry, where early studies began to quantify properties such as surface tension, cohesion, and interfacial energetics. A landmark contribution came with mid-20th century investigations—exemplified by a 1951 Nature report—that established the unusually high surface tension of liquid metals and highlighted their nonwetting, high-energy interfaces, distinguishing them fundamentally from conventional liquids.^51^

Prior to the widespread adoption of gallium-based systems, research on liquid metals remained largely confined to thermophysical characterization and metallurgical contexts, with limited exploration of their interfacial dynamics under ambient conditions. It was only in later decades, particularly with the emergence of Ga-based alloys such as EGaIn and Galinstan, that attention shifted toward their unique combination of fluidity, conductivity, and surface oxide formation, opening new directions in interfacial science. These foundational physicochemical insights—especially into oxide skin formation, surface energy modulation, and interfacial stability—would ultimately enable pioneers to harness liquid metals as functional inks, laying the groundwork for their transformation into key materials for printable and reconfigurable electronics.

It was first shown that eutectic gallium–indium (EGaIn) behaves as a surface-dominated, yield-stress fluid, where a nanometric oxide skin stabilizes otherwise fluid metal into moldable, shape-retaining structures at room temperature.^52^ By identifying a critical surface stress governing flow and stability, this work established that liquid metal shaping can be controlled through interfacial physics rather than bulk solidification. The same team demonstrated that the oxide-stabilized interface enables reproducible microscale electrical contacts under ambient conditions, overcoming the instability and toxicity limitations of mercury-based systems (Figure 4B­(i)).^53^ They later revealed that the mechanical response of liquid metals is governed by a strain-history-dependent oxide skin, introducing surface-controlled yielding and time-evolving elasticity that redefine these materials as surface-governed, non-Newtonian systems·^54^

Building on this paradigm, a subsequent foundational study introduced liquid metal marbles: nanoparticle-coated droplets that behave as nonwetting, reconfigurable, electronically active soft particles. This architecture created mobile metal–semiconductor junctions that could form adaptive, noncontact circuits while reducing issues of corrosion, adhesion, and rigid interfacing.^55^ Another key advance demonstrated that the oxide layer itself can be electrochemically programmed, where low applied potentials drive in situ oxidation and reduction to reversibly tune surface tension from ∼ 500 mN/m to near zero (Figure 4B­(ii,iii)). This capability effectively transforms interfacial energy into a dynamic control parameter for shape, flow, and function.^56^ Complementary to static electrochemical control of surface tension, it has also been shown that liquid metal fluidity can arise from a dynamic oxidation–dissolution cycle, enabling superwetting and continuous penetration through porous media via kinetically sustained low interfacial resistance (Figure 4B­(iv)).^57^ Taken together, these advances establish a unifying principle: liquid metals are not passive conductors but programmable interfacial systems, where surface chemistry, mechanics, and electrochemistry converge—laying the physicochemical foundation for their emergence as a transformative platform in printable and reconfigurable electronics.^58^

Liquid metals have moved far beyond their traditional role as simple conductors and now act as active, versatile platforms for printing functional materials and devices (Figure 4B­(vii,viii)). It is now possible to directly generate atomically thin, highly transparent conductive films from liquid metal systems under ambient, vacuum-free conditions, opening a pathway toward scalable and low-cost fabrication of next-generation optoelectronics.^59^ At the same time, liquid metals can be printed onto porous and deformable substrates to form permeable, stretchable conductive networks, where the material self-organizes into architectures that maintain electrical integrity even under extreme mechanical strain—offering breathable, wearable electronics without sacrificing performance.^60^ This overcomes long-standing interfacial limitations associated with insulating particle shells and enables multilayer, self-passivated electronic architectures with robust interconnects and reliable operation in real-world conditions.^61^ Beyond planar systems printed from room-temperature liquid metals like EGaIn, it is now possible to directly print free-standing, high-aspect-ratio structures from Field’s metal without supports or postprocessing, where interfacial tension and rapid solidification guide the formation of stable geometries (Figure 4B­(vi)).^62^

## 4 ■ Grand Challenges: a Call for Physicochemical Innovation

The remaining barriers in printed devices and additive manufacturing should not be understood only as issues of technology maturity, machine speed, or cost reduction. They also concern the translation of laboratory-scale breakthroughs into scalable manufacturing ecosystems, where reproducibility, long-term operational stability, process integration, standardization, and commercially viable production pathways become decisive. The deeper lesson from the breakthroughs discussed above is that transformative printing platforms often emerge only after decades of fundamental scientific understanding of light–matter interaction, capillary flow, polymerization thresholds, interfacial forces, phase transformation, thermal transport, or reaction kinetics, that has matured into controllable manufacturing principles. In this sense, the next leap in printable devices will not come simply from building better printers, but from discovering and implementing new physical chemistry that allows matter to be patterned, transformed, integrated, and stabilized with predictive control across both laboratory demonstrations and scalable production environments.

### 4.1 Liquid-Phase Challenges

Most of today’s scalable printed electronics and functional devices still rely on solution- and liquid-based methods, including droplet, jet, extrusion, capillary, aerosol, and meniscus-guided processes. These approaches have brought printing close to the vision of low-cost, flexible, and distributed manufacturing, but they remain strongly constrained by the physics and chemistry of fluids. Ink stability, rheology, wetting, evaporation, particle assembly, drying, coffee-ring flows, nozzle clogging, substrate adhesion, sintering, and postprint curing are still often optimized empirically rather than predicted from first principles. These challenges appear differently across methods: droplet-based printing is vulnerable to evaporation-driven nonuniformity and nozzle instability; extrusion and screen printing depend on shear-thinning behavior, yield stress, and binder chemistry; aerosol and electrohydrodynamic approaches add further complexity through gas-flow or electric-field-driven transport and jet stability; high-resolution photopolymerization methods depend on threshold chemistry, energy localization, and nanoscale polymer network formation.

Here, physical chemistry has a decisive role to play. The field needs predictive frameworks that connect ink composition to flow, spreading, drying, microstructure, and final function. It also needs a deeper understanding of how colloidal stability, capillary forces, interfacial energy, solvent evaporation, phase separation, polymerization kinetics, charge transport, and material–substrate coupling collectively define printability and performance. With smart optimization and closed-loop process control, these complex, multivariable systems could move beyond trial-and-error toward autonomous formulation design and real-time correction. The challenge is therefore not only to print finer or faster, but to make liquid-based printing a programmable physicochemical process.

### 4.2 Inkless Printing Frontiers

While the advances discussed so far have been largely driven by solution- and liquid-based printing, a parallel and conceptually distinct direction is emerging. In solution-free printing, materials are no longer carried by inks but are delivered or even formed directly from vapor, solid, or reactive precursor states. This removes the need for solvents and additives, opening the possibility of printing materials in more pristine forms, with direct control over composition, phase, and nanostructure as they emerge on the substrate. In principle, this is a powerful reimagining of printing: not only deposition, but the programming of matter formation in space and time.

Yet solution-free printing remains far from becoming a widely scalable manufacturing paradigm. Its reliance on specialized environments, limited material versatility, high temperature or vacuum requirements, and challenges in achieving large-area, high-throughput patterning have kept it closer to a niche frontier than a mainstream platform. More fundamentally, without the stabilizing and confining role of solvents, transport, reaction, and assembly occur in highly dynamic nonequilibrium environments governed by vapor-phase diffusion, interfacial thermodynamics, precursor chemistry, heat and mass transfer, and energy–matter interactions. This makes it difficult to control where materials form, how phases evolve, and how microstructures emerge. If physical chemistry can provide predictive control over nucleation, reaction kinetics, interfacial growth, and in situ phase formation, solution-free methods could evolve from precise but specialized tools into more realistic routes for lower-cost and high-performance manufacturing.

### 4.3 From Patterns to Systems

Beyond the immediate challenges of printability and pattern formation, a deeper set of gaps separates printed 2D and 3D technologies from their broader manufacturing promise. Printed structures must retain their function under bending, stretching, heat, humidity, oxidation, light exposure, chemical stress, repeated operation, and long-term environmental conditions. Yet many current demonstrations remain proof-of-concept studies, with limited durability data and few standardized accelerated-aging protocols. Future printed materials must therefore be designed not only for high initial performance, but for functional durability, process tolerance, reproducibility, and large-area uniformity.

This challenge becomes even larger when printed layers are integrated into complete devices. Future systems must combine active materials with interconnects, power sources, encapsulation layers, readout circuits, wireless modules, energy storage or harvesting units, and hybrid electronic components, without losing the flexibility, conformability, or sustainability that make printing attractive. These are not merely engineering afterthoughts. They are grand physical chemistry problems involving degradation pathways, interfacial mechanics, adhesion, polymer–particle interactions, ion and charge transport, encapsulation chemistry, and heterogeneous interfaces. If physical chemistry can clarify how printed materials age, fail, bond, conduct, and interact across interfaces, printing can move from isolated functional patterns toward reliable, connected, and adaptive devices.

### 4.4 Smart and Circular Scale-Up

A final grand challenge is to transform printing from a demonstration-driven field into a certifiable, sustainable, and intelligent manufacturing science. Current roadmaps repeatedly point to the need for predictive models linking design, process parameters, thermal and chemical history, microstructure, defects, and final function. The field also lacks mature approaches for voxel-level control, where composition, porosity, crystallinity, conductivity, stiffness, or chemical functionality can be programmed locally within a printed object. Real-time monitoring, digital twins, defect prevention, postprocessing reduction, qualification, standardization, and material databases will be essential if printed devices are to be trusted at scale.

Equally important is the sustainability promise of printing. The field cannot claim to democratize manufacturing while relying on toxic solvents, expensive feedstocks, energy-intensive postprocessing, nonrecyclable substrates, or poorly understood end-of-life pathways. Green solvents, water-based inks, low-temperature sintering, recyclable binders, biodegradable substrates, materials recovery, and cradle-to-grave design must become central scientific targets rather than peripheral considerations. The distributed and digitally driven nature of printable electronics may also provide a natural pathway for adopting emerging digital product passport concepts, where materials, processing history, performance metrics, repairability, and end-of-life information can be linked to individual devices to enhance traceability, supply chain transparency, and circular resource management. In this future, AI can help navigate complex formulation and processing spaces, but it cannot replace physical chemistry. The real opportunity lies in physics-informed AI: models grounded in thermodynamics, kinetics, transport, interfacial science, and degradation mechanisms. Such integration could allow printing systems to sense, learn, correct, and certify their own processes in real time.

Taken together, these challenges define a clear call to physical chemistry scientists. The future of printing will depend on our ability to understand and control matter under transient, confined, reactive, and nonequilibrium conditions. If this science can be advanced, printing may no longer be viewed simply as a cheaper way to fabricate devices, but as a new paradigm for manufacturing functional matter on demand—reliable, sustainable, adaptive, and accessible wherever it is needed.
